# Beyond Access: A Multi-domain Framework for Assessing Digital Health Underutilisation in Non-communicable Disease Care in Low- and Middle-Income Countries

**DOI:** 10.7759/cureus.111833

**Published:** 2026-06-30

**Authors:** Sagarika Duggirala, Satyavani Kumpatla, Vijay Viswanathan, D S Prasad

**Affiliations:** 1 Community Medicine, Great Eastern Medical School and Hospital, Srikakulam, IND; 2 Research, M.V. Hospital for Diabetes and Diabetes Research Centre, Chennai, IND; 3 Diabetes and Endocrinology, M.V. Hospital for Diabetes and Diabetes Research Centre, Chennai, IND; 4 Cardiology, Sudhir Heart Centre, Berhampur, IND

**Keywords:** chronic disease management, diabetes mellitus, digital divide, digital health, ehealth literacy, hypertension, implementation framework, low- and middle-income countries, non-communicable diseases, telemedicine

## Abstract

Digital-health technologies are increasingly being incorporated into the management of non-communicable diseases (NCDs); however, meaningful utilisation remains inconsistent in many low- and middle-income countries (LMICs) despite expanding digital infrastructure and telemedicine availability. Existing literature has largely focused on disease-specific interventions or individual digital-health tools, while structured, implementation-oriented frameworks for assessing digital-health underutilisation across diverse NCD settings remain limited.

This technical report presents a practical multi-domain framework for assessing digital-health underutilisation in NCD care within LMIC settings. The framework conceptualises utilisation as a multidimensional process influenced by structural access, individual capability, behavioural readiness, and health-system factors. It incorporates assessment domains related to device and internet access, digital and eHealth literacy, attitudes toward digital-health use, contextual barriers, and integration within routine healthcare pathways. The report additionally outlines a stepwise implementation workflow intended to support structured assessment across outpatient, community, and chronic disease-care settings. The framework was informed by practical operational experience derived from structured outpatient digital-health assessment implemented within a tertiary-care setting in Southern India and is designed to remain adaptable across a broader range of NCD-care environments.

Digital-health underutilisation in LMICs reflects a complex interaction of technological, behavioural, literacy-related, and health-system determinants rather than limitations in access alone. The framework presented in this report provides a practical and reproducible implementation-oriented approach for systematically evaluating barriers to digital-health utilisation and may support context-sensitive strategies for strengthening digital-health integration within NCD care.

## Introduction

Digital-health technologies have emerged as an important component of contemporary healthcare systems, particularly in the management of non-communicable diseases (NCDs) such as diabetes, hypertension, cardiovascular disease, and chronic respiratory disorders [1,2]. Tools including mobile-health applications, teleconsultation platforms, short message service (SMS) reminders, remote monitoring systems, and patient portals have demonstrated potential to improve treatment adherence, facilitate continuity of care, support self-management, and enhance patient engagement across diverse clinical settings [3-5].

Over the past decade, global and national initiatives have accelerated the integration of digital technologies into healthcare delivery [1,6]. In India, the expansion of digital-health infrastructure through initiatives such as the National Digital Health Mission and the Ayushman Bharat Digital Mission has created new opportunities for strengthening chronic disease-care pathways and improving healthcare accessibility [6]. The COVID-19 pandemic further accelerated adoption of telemedicine and digitally enabled models of care, particularly for patients requiring long-term follow-up and uninterrupted access to healthcare services [4,7].

Despite the increasing availability of digital-health infrastructure, utilisation of these technologies remains inconsistent, particularly in low- and middle-income countries (LMICs). This mismatch between availability and meaningful use represents a critical implementation gap. In the present report, digital-health underutilisation refers broadly to suboptimal engagement with available digital-health services despite potential opportunities for use. The concept extends beyond simple non-adoption or limited access and may also encompass low-frequency use, discontinuation, or incomplete engagement resulting from behavioural, literacy-related, contextual, or health-system barriers. Factors contributing to underutilisation are multifactorial and include limited digital literacy, socioeconomic constraints, variable access to devices and internet connectivity, behavioural resistance, language barriers, and health system-level limitations [8-10]. In many LMIC settings, including rural and semi-urban regions of South Asia, these challenges persist despite the increasing penetration of mobile technologies and the expansion of digital-health ecosystems [10].

Existing literature has largely focused on disease-specific applications, individual digital interventions, or telemedicine models within selected clinical contexts [2-5]. However, there remains a relative paucity of structured, implementation-oriented frameworks that systematically assess digital-health utilisation across multiple domains relevant to NCD care in LMIC settings. In particular, there is limited availability of operational frameworks that integrate individual capability, behavioural readiness, technological access, and health-system factors into a unified assessment model applicable to routine clinical and public-health settings [11].

Addressing this gap is important not only for understanding current utilisation patterns but also for informing scalable and contextually appropriate digital-health strategies. A structured methodological framework can facilitate standardised assessment, improve comparability across studies, and support evidence-based policy and programme development in resource-constrained environments [11].

The framework presented in this technical report was informed by practical implementation experience derived from a structured outpatient digital-health assessment model previously operationalised in a tertiary-care setting in Southern India [12]. Building on this applied experience, together with broader LMIC digital-health evidence, the present report proposes a practical, multi-domain framework for assessing digital-health underutilisation in NCD care. The objective is to provide a practical and adaptable approach that can assist researchers, clinicians, programme managers, and public-health practitioners in systematically identifying barriers to digital-health utilisation and informing targeted implementation strategies across diverse LMIC settings.

## Technical report

Conceptual basis and framework structure

Digital-health underutilisation in NCD care is increasingly recognised as a multidimensional implementation challenge rather than a simple consequence of limited technological availability alone [1,2]. In many LMIC settings, expanding access to smartphones, internet services, and telemedicine platforms has not consistently translated into sustained or meaningful engagement with digital-health services within routine healthcare delivery [9]. This gap reflects the interaction of overlapping structural, individual, behavioural, and health-system determinants influencing digital-health utilisation across diverse clinical and community settings [8-10].

The proposed framework was developed through the conceptual synthesis of practical implementation experience and published digital-health literature. Practical experience was informed by structured outpatient assessment approaches evaluating digital-health utilisation, eHealth literacy, behavioural readiness, and perceived barriers within routine chronic disease care. These operational components were iteratively integrated with implementation determinants identified in the literature and relevant global guidance pertaining to digital-health implementation in NCD care within LMIC settings. The framework is not intended as a new measurement scale and is not a direct reproduction of any previously published model. Rather, it represents an implementation-oriented conceptual adaptation designed to facilitate the systematic assessment of digital-health underutilisation across diverse NCD-care settings. The framework was not derived through a formal systematic review, scoping review, Delphi process, stakeholder workshop, or psychometric framework-development exercise. Consequently, it should be regarded as a conceptual implementation framework intended to support structured assessment and future evaluation, with formal validation and stakeholder-informed refinement representing important areas for future research.

**Figure 1:**
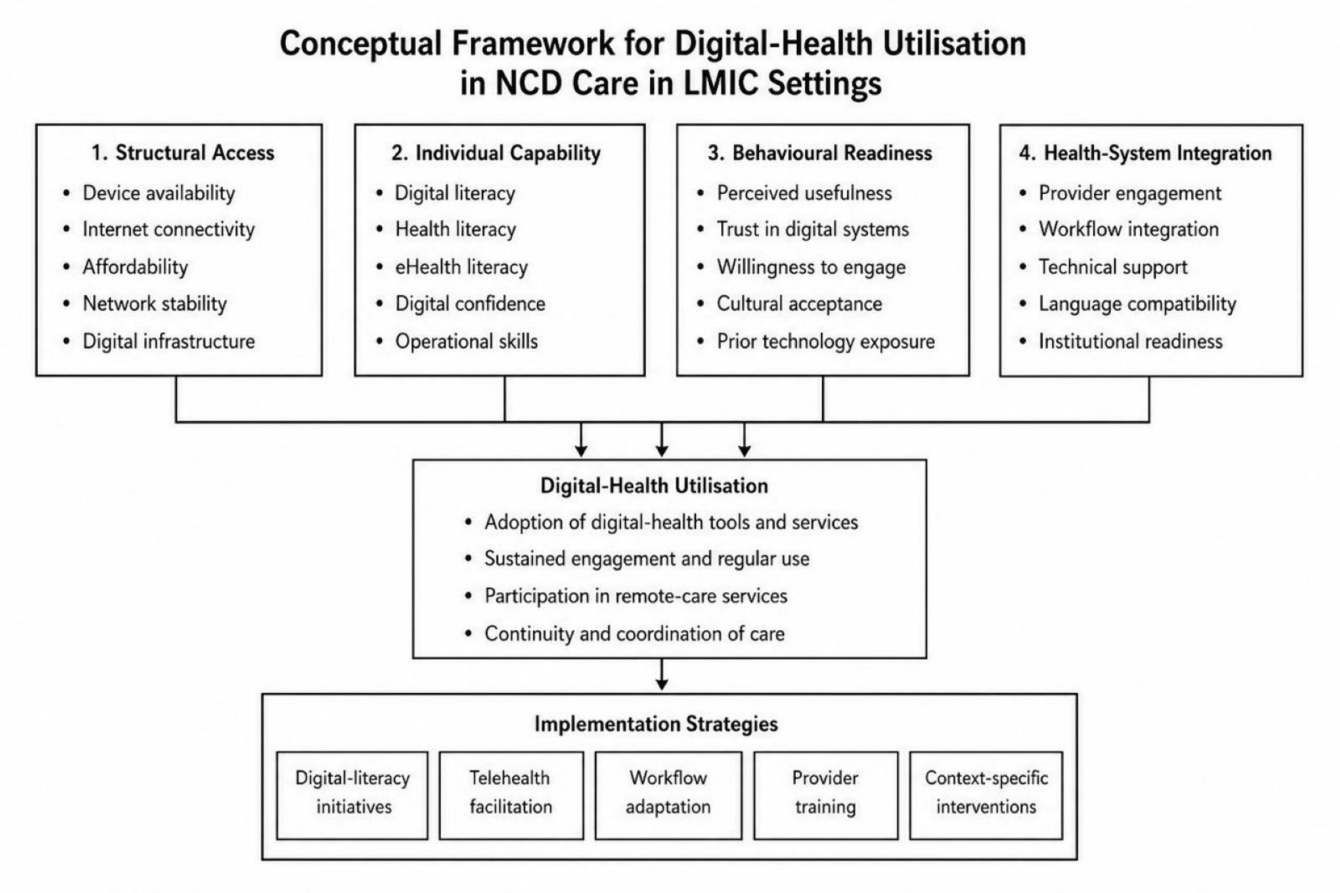
Multi-Domain Framework for Assessing Digital-Health Utilisation in NCD Care Conceptual illustration of the multidomain framework demonstrating interactions between structural access, individual capability, behavioural readiness, and health-system integration influencing digital-health utilisation across routine non-communicable disease-care settings. Abbreviations: NCD, non-communicable disease; LMIC, low- and middle-income countries.

The framework presented in this report adopts a multi-domain implementation-oriented approach that conceptualises utilisation behaviour as the outcome of interacting determinants operating across four interrelated domains: structural access, individual capability, behavioural readiness, and health-system integration. The conceptual relationship among the major framework domains influencing digital-health utilisation is illustrated in Figure 1.

Core framework domains and illustrative assessment indicators are summarised in Table 1.

**Table 1:** Core Domains and Illustrative Components of the Proposed Digital-Health Utilisation Framework Abbreviations: eHEALS, eHealth Literacy Scale; LMICs, low- and middle-income countries; NCDs, non-communicable diseases.

Domain	Key Components	Illustrative Assessment Indicators
Structural Access	Device ownership, internet connectivity, affordability, network reliability	Smartphone access, internet availability, frequency of connectivity interruptions
Individual Capability	Digital literacy, health literacy, eHealth literacy, digital confidence	eHEALS score, ability to navigate digital platforms, confidence in using digital tools
Behavioural Readiness	Perceived usefulness, trust, willingness to engage, cultural acceptance	Likert-scale responses, attitudes toward teleconsultation, preference for in-person care
Health-System Integration	Provider engagement, workflow integration, technical support, institutional readiness	Availability of telehealth services, provider support, local-language compatibility
Utilisation Outcomes	Adoption, sustained engagement, modality diversity, continuity of care	Teleconsultation use, mobile-health application use, frequency of digital-health engagement

The structural-access domain includes factors related to availability of digital devices, internet connectivity, affordability of services, network reliability, and access to digital infrastructure. Although access is often regarded as a primary prerequisite for digital-health adoption, evidence from LMIC settings suggests that technological access alone may be insufficient to ensure meaningful utilisation [9]. Intermittent connectivity, shared device usage, financial limitations, and variable digital infrastructure may continue to affect consistency of engagement in resource-constrained environments.

The individual-capability domain focuses on digital literacy, health literacy, and eHealth literacy, including the ability to search, interpret, evaluate, and apply health-related information obtained through electronic platforms [7,8]. Standardised tools such as the eHealth Literacy Scale (eHEALS) may facilitate reproducible assessment of this dimension [7]. In many LMIC populations, particularly among older adults and individuals with limited formal education, inadequate digital confidence and reduced eHealth literacy may substantially influence utilisation patterns despite the availability of digital-health services [9].

The behavioural-readiness domain encompasses attitudinal and behavioural determinants, including perceived usefulness of digital-health tools, trust in digital platforms, willingness to engage with remote-care models, and preferences regarding face-to-face consultation. Behavioural readiness may additionally be shaped by prior exposure to technology, sociocultural context, family support systems, and previous healthcare experiences. These factors may influence sustained engagement even where structural access is available.

The health-system integration domain incorporates contextual and institutional determinants, including provider engagement, workflow integration, language compatibility, technical support systems, institutional readiness, and local implementation constraints. Fragmented digital ecosystems, limited interoperability, and inadequate provider training may contribute to suboptimal utilisation despite the expansion of digital-health initiatives [2,11]. In many LMIC settings, the effectiveness of digital-health implementation, therefore, remains closely linked to broader health-system preparedness and contextual adaptation.

Importantly, underutilisation of digital-health technologies may persist even in populations with reasonable access to mobile devices or internet connectivity. Prior evidence from South Asian and other LMIC settings suggests that limited digital confidence, low eHealth literacy, behavioural resistance, inadequate institutional support, and a preference for conventional consultation models continue to influence patterns of utilisation [7-9]. These observations support the need for implementation-oriented frameworks that extend beyond technology deployment alone and instead incorporate behavioural, contextual, and system-level determinants of engagement.

The framework presented in this report was informed by practical outpatient implementation experience involving structured assessment of digital-health utilisation, literacy, behavioural readiness, and perceived barriers within a tertiary-care setting in Southern India [12]. While initially operationalised within a diabetes-care context, the conceptual structure is intended to remain adaptable across a broader spectrum of chronic NCD conditions requiring long-term follow-up, continuity of care, behavioural modification, and patient engagement. A sample operational assessment checklist derived from the framework is presented in Table 2 to support structured implementation and practical application across diverse healthcare settings.

**Table 2:** Sample Operational Assessment Checklist for Digital-Health Utilisation in NCD Care Scoring examples are illustrative and may be adapted according to local implementation requirements, healthcare settings, study objectives, target populations, and data-collection methods. Respondents, units of analysis, recall periods, and scoring thresholds should be defined according to the specific implementation context and evaluation objectives. Abbreviations: eHEALS, eHealth Literacy Scale; NCD, non-communicable disease.

Framework Domain	Example Assessment Variable	Suggested Operational Assessment Method	Illustrative Scoring/Interpretation
Structural Access	Smartphone ownership	Self-report questionnaire	Yes = 1; No = 0
	Internet availability	Household internet/mobile data access assessment	Regular access = 2; Intermittent = 1; No access = 0
	Network reliability	Patient-reported connectivity stability	Stable = 2; Variable = 1; Poor = 0
	Affordability of digital services	Perceived affordability of internet/device use	Affordable = 2; Partially affordable = 1; Not affordable = 0
Individual Capability	Digital literacy	Structured assessment of basic smartphone/app use	Adequate = 2; Limited = 1; Inadequate = 0
	eHealth literacy	Standardised tools such as eHEALS	Higher scores indicate better eHealth literacy
	Confidence in digital-health use	Likert-scale assessment	High = 2; Moderate = 1; Low = 0
	Ability to interpret online health information	Structured questionnaire/interview	Adequate = 2; Partial = 1; Poor = 0
Behavioural Readiness	Perceived usefulness of digital-health tools	Likert-scale assessment	Positive = 2; Neutral = 1; Negative = 0
	Willingness to use teleconsultation	Direct patient-response assessment	Willing = 2; Uncertain = 1; Unwilling = 0
	Preference for in-person consultation	Behavioural preference assessment	Strong preference may indicate reduced digital readiness
	Trust in digital platforms	Structured attitudinal assessment	High = 2; Moderate = 1; Low = 0
Health-System Integration	Provider encouragement for digital-health use	Patient-reported provider engagement	Consistent = 2; Occasional = 1; Absent = 0
	Availability of technical support	Institutional support assessment	Adequate = 2; Limited = 1; None = 0
	Integration into routine care pathways	Clinical workflow assessment	Integrated = 2; Partial = 1; Not integrated = 0
	Language compatibility of digital platforms	User-reported accessibility	Adequate = 2; Partial = 1; Poor = 0

Measurement domains and operational indicators

Operationalisation of the framework is based on a systematic assessment of factors influencing digital-health utilisation across four interrelated domains: structural access, individual capability, behavioural readiness, and health-system integration. The objective is not merely to determine whether digital-health tools are available, but to evaluate the extent to which individuals are able, willing, and supported to engage with such technologies within routine NCD care.

Digital-health utilisation can be evaluated as a binary outcome (use versus non-use within a defined period) or as a multidimensional construct reflecting the frequency, diversity, and continuity of engagement with digital-health services. Assessment may encompass a range of modalities, including mobile-health applications, teleconsultation platforms, SMS-based communication, wearable-device integration, remote monitoring systems, online support networks, and web-based educational resources. Evaluation across multiple modalities provides a broader understanding of utilisation patterns and implementation gaps across different clinical and public-health contexts.

Each framework domain is supported by structured indicators, standardised public-health surveillance principles, and context-appropriate measurement instruments [13]. Structural-access indicators include device ownership, smartphone availability, internet connectivity, affordability, and network reliability. Individual capability can be evaluated using validated tools such as the eHEALS, together with assessment of digital confidence and basic operational skills [7]. Behavioural-readiness indicators include perceptions of usefulness, trust in digital-health systems, willingness to engage with remote-care models, and preferences regarding conventional in-person consultation.

Health-system integration may be assessed through indicators related to provider engagement, institutional integration of digital-health platforms, availability of technical support, language compatibility, and continuity of digital-care pathways. Contextual assessment may additionally identify barriers related to geography, sociocultural influences, workforce limitations, and healthcare accessibility. Selection of measurement instruments should consider reliability, construct validity, and contextual appropriateness, particularly in heterogeneous LMIC populations [14,15].

The framework is intended to support implementation-oriented assessment rather than disease-specific prediction. By integrating structural, behavioural, literacy-related, and health-system measures, the proposed approach facilitates a broader evaluation of meaningful digital-health engagement beyond simple access-based assessment. Such multidomain evaluation may help clinicians, researchers, and programme managers identify implementation barriers and prioritise context-sensitive strategies for improving digital-health utilisation across routine NCD-care settings.

Analytical and implementation considerations

The framework supports both descriptive and analytical applications across diverse NCD-care settings. In observational and implementation-oriented studies, multivariable analytical approaches may be used to identify factors associated with digital-health utilisation while incorporating variables from multiple framework domains.

Particular attention should be paid to potential collinearity between socioeconomic status, educational attainment, age, digital literacy, and eHealth literacy, as these variables may demonstrate substantial overlap in LMIC populations [7-9]. Analytical approaches should therefore prioritise conceptual and contextual relevance in addition to statistical considerations, particularly in heterogeneous healthcare settings.

The framework additionally permits stratified assessment across population subgroups defined by age, gender, residence, educational status, socioeconomic profile, or disease category. Such subgroup analyses may help identify populations at greater risk of digital exclusion and may provide insight into context-specific barriers influencing utilisation patterns in LMIC environments.

Beyond binary classification of utilisation, the framework also supports exploratory evaluation of graded digital-health engagement, including frequency, continuity, and diversity of modality use. This may facilitate broader assessment of sustained engagement with digital-health services across different chronic disease-care pathways.

Importantly, the proposed framework is intended primarily as an implementation-oriented assessment model rather than a disease-specific predictive algorithm. Its principal utility lies in facilitating structured evaluation of utilisation barriers, supporting context-sensitive intervention planning, and improving comparability across digital-health implementation studies conducted in diverse NCD-care settings.

Stepwise implementation workflow

Implementation of the framework involves a structured and context-sensitive workflow that may be adapted across different NCD-care environments. The initial step includes defining the target population and implementation setting, which may involve outpatient NCD clinics, community-based screening programmes, telemedicine follow-up services, or integrated chronic disease-management pathways.

The second step involves selection of relevant framework domains and contextual variables according to local implementation requirements. This process should be informed by consideration of stakeholder perspectives, including those of patients, caregivers, clinicians, programme managers, and other relevant implementation partners, to ensure contextual relevance and feasibility. While the four core domains remain central to the framework, additional factors such as language barriers, rural accessibility, caregiver involvement, local digital-service availability, and health-system constraints may also be incorporated depending on the healthcare setting. Context-specific prioritisation of domains and variables may help improve the practical applicability of the framework across diverse NCD-care environments.

The third step includes identification of appropriate measurement instruments and data-collection methods. Validated tools such as eHEALS may be used for assessment of eHealth literacy, while structured questionnaires and contextual indicators may support evaluation of behavioural, technological, and health-system factors [7]. Depending on local feasibility and literacy considerations, data collection may be conducted through interviewer-administered surveys, digital questionnaires, or hybrid approaches.

The subsequent step involves assessment of utilisation patterns across different digital-health modalities, including teleconsultation services, mobile-health applications, messaging systems, remote monitoring platforms, and educational resources. Simultaneous assessment of perceived barriers and facilitators may help contextualise utilisation behaviour and identify implementation gaps.

Following assessment, findings may be stratified to identify vulnerable subgroups and priority areas for intervention. Potential implementation barriers may include limited digital literacy, inadequate structural access, reduced behavioural readiness, or insufficient institutional support. The final step involves translation of findings into context-sensitive implementation strategies such as digital-literacy initiatives, workflow integration, provider training, language adaptation, telehealth facilitation, and strengthening of digital-health infrastructure.

**Figure 2:**
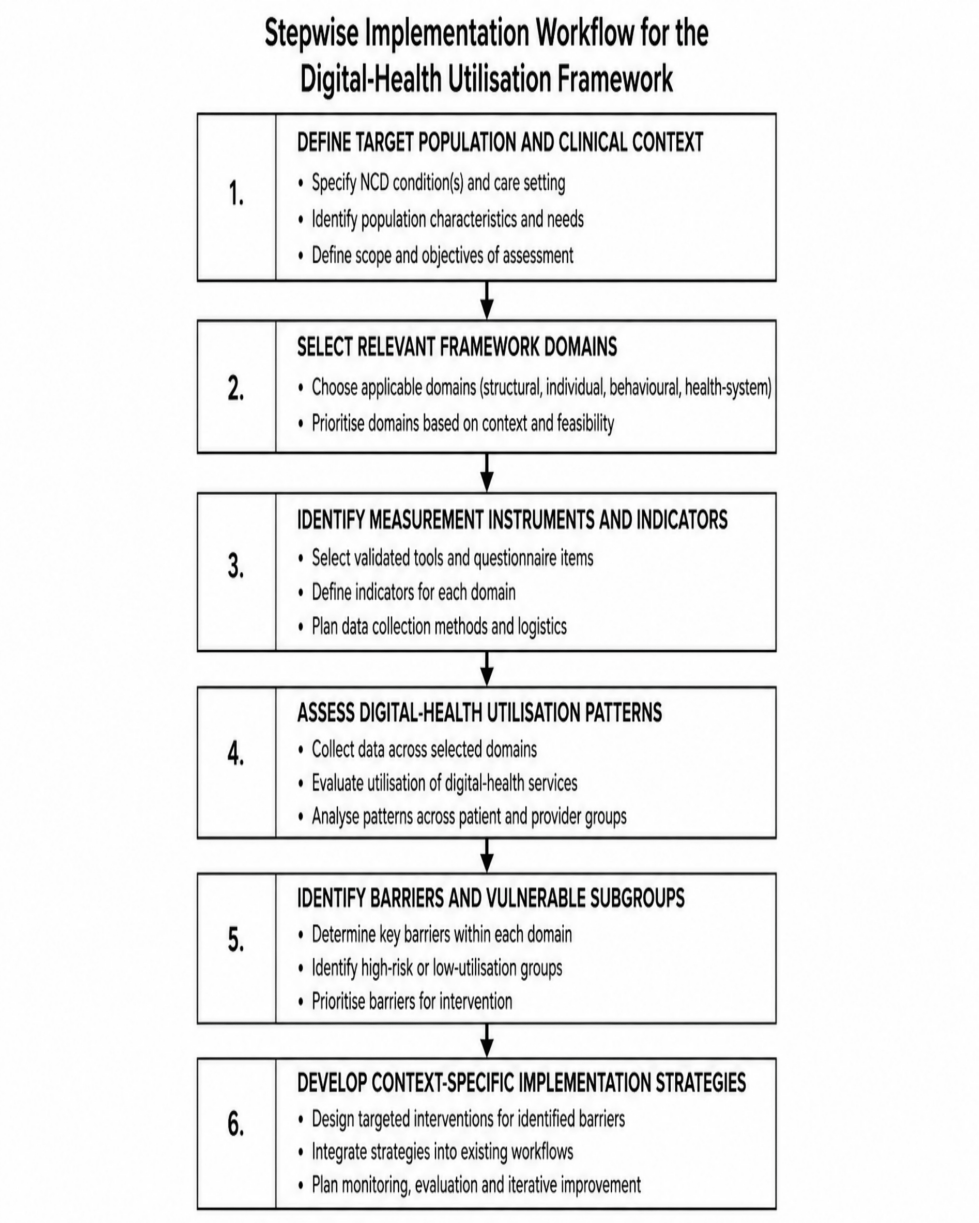
Stepwise Implementation Workflow for the Digital-Health Utilisation Framework Illustrative stepwise workflow demonstrating implementation of the framework across routine non-communicable disease care settings, including domain assessment, barrier identification, contextual analysis, utilisation assessment, and development of targeted implementation strategies.

This workflow is intended to provide a practical and adaptable implementation pathway for systematic assessment of digital-health underutilisation across diverse NCD-care settings in LMICs. An illustrative stepwise workflow for implementation of the framework across NCD-care settings is presented in Figure 2.

Illustrative application across NCD settings

The applicability of the proposed framework is supported by its prior operational use within a structured tertiary-care outpatient setting in Southern India, where digital-health utilisation was assessed alongside measures of eHealth literacy, behavioural readiness, and perceived barriers [12]. In that implementation context, utilisation patterns appeared to be influenced by the interaction of structural, behavioural, literacy-related, and contextual determinants rather than by technological availability alone.

The implementation experience informing this framework included an assessment of digital-health modalities, such as mobile-health applications, teleconsultation services, and online communication tools, within routine chronic disease-care pathways. Structured evaluation of eHealth literacy and attitudinal readiness provided additional insight into factors influencing engagement with digital-health services.

Although the original implementation setting primarily involved diabetes care, the broader framework is intended to remain adaptable across other NCD settings, including hypertension, cardiovascular disease, chronic respiratory disease, and multimorbidity care pathways. Several implementation challenges identified in the original setting - including limited digital literacy, behavioural resistance, variable connectivity, and fragmented health-system integration - are similarly encountered across diverse chronic disease settings in LMIC environments.

The present report does not reproduce dataset-specific findings from the earlier implementation study [12]. Rather, the prior operational experience is intended to illustrate how the framework may be applied within routine clinical and public-health settings to support structured assessment of digital-health utilisation and identify context-specific implementation barriers.

Operational assessment checklist and scoring template

To improve operational applicability and reproducibility, the framework incorporates a structured assessment checklist and illustrative scoring template for evaluation of digital-health utilisation across NCD-care settings. The operational checklist presented in Table 2 provides example indicators across the major framework domains, including structural access, individual capability, behavioural readiness, and health-system integration.

The checklist is intended to support systematic and context-sensitive assessment of barriers influencing digital-health engagement using structured implementation-oriented assessment approaches adapted from public-health surveillance principles [13]. Although illustrative scoring approaches are included to facilitate operational use, the framework is not intended to function as a rigid quantitative scoring system. Rather, the checklist and scoring template are designed to support flexible implementation-oriented assessment across diverse healthcare settings where contextual priorities, population characteristics, and digital-health infrastructure may differ substantially.

The proposed operational template may assist clinicians, researchers, programme managers, and policymakers in identifying vulnerable populations, prioritising implementation barriers, and planning context-specific interventions. Potential applications may include baseline assessment of digital-health readiness, subgroup evaluation, monitoring of implementation gaps, and comparative assessment across different NCD-care settings. The framework may additionally support future implementation research aimed at refining operational indicators and evaluating digital-health integration strategies in resource-constrained healthcare environments.

## Discussion

Existing digital-health and implementation frameworks

Several conceptual and implementation-oriented frameworks have previously been used to evaluate the adoption and utilisation of digital-health technologies. Models such as the Technology Acceptance Model (TAM) and the Unified Theory of Acceptance and Use of Technology (UTAUT) have primarily focused on behavioural determinants influencing technology acceptance, including perceived usefulness, ease of use, social influence, and behavioural intention [16,17]. These frameworks have contributed substantially to understanding factors affecting adoption of digital platforms across healthcare and non-healthcare settings.

Implementation-science frameworks, such as the Consolidated Framework for Implementation Research (CFIR), have additionally provided broader approaches for evaluating implementation processes across healthcare systems, organisational environments, and service-delivery contexts [18]. Similarly, the World Health Organization’s digital-health strategy emphasises the importance of health-system integration, interoperability, governance, workforce preparedness, and context-sensitive implementation of digital-health interventions [1].

Although these frameworks provide important conceptual and implementation guidance, several challenges remain when applying them within routine NCD-care settings in LMIC environments. Many implementation models are operationally complex, resource intensive, or oriented primarily toward organisational implementation research rather than pragmatic assessment within routine outpatient or community-based care settings. In addition, digital-health utilisation in LMIC populations is often influenced by overlapping structural, behavioural, literacy-related, and contextual determinants that may not be assessed simultaneously within a single operational framework.

The framework proposed in the present report is intended to complement rather than replace existing implementation and technology-adoption models. Its primary emphasis lies in providing a practical, multidomain, and implementation-oriented assessment approach adaptable to routine NCD-care settings in resource-constrained environments. By integrating structural access, individual capability, behavioural readiness, and health-system integration within a single operational framework, the model aims to support structured assessment of utilisation barriers while remaining sufficiently flexible for application across diverse chronic disease-care pathways and heterogeneous LMIC healthcare settings.

Global evidence and post-COVID digital-health expansion

Digital-health technologies are increasingly being incorporated into chronic NCD management across diverse healthcare settings. Emerging evidence suggests that meaningful utilisation depends not only on technological access but also on literacy, behavioural readiness, and integration within routine care pathways [3-6]. Multicomponent digital-health approaches, including telemonitoring, mobile-health applications, and remote consultation systems, have demonstrated potential utility in improving treatment adherence, continuity of care, and patient engagement in chronic disease management [3-6].

In India, digitally enabled care models have shown applicability in supporting long-term diabetes management and remote follow-up, particularly when combined with clinician engagement and structured monitoring systems [3-6]. Recent evidence from a randomised controlled trial among cardiac patients additionally demonstrated that multicomponent digital-health and telemonitoring interventions were associated with improved hypertension and glycaemic control, supporting the broader applicability of digitally enabled chronic disease-management strategies across NCD settings [19].

The COVID-19 pandemic further accelerated adoption of digital-health technologies globally. During periods of restricted mobility and healthcare-system disruption, telemedicine evolved from a supplementary healthcare tool into an important mechanism for maintaining continuity of chronic disease care [11]. Remote consultations, telephonic follow-up, electronic prescription systems, and digital communication platforms facilitated ongoing interaction between healthcare providers and patients while reducing dependence on in-person visits [4,11].

Several digital-health adaptations introduced during the pandemic have subsequently persisted within routine healthcare practice, contributing to the emergence of hybrid models integrating conventional and digital-care delivery [11]. At the same time, the rapid expansion of digital-health use during COVID-19 highlighted substantial disparities in digital readiness, literacy, access, and utilisation, particularly in LMIC settings. Collectively, these observations reinforce the importance of implementation-oriented frameworks capable of systematically evaluating barriers influencing meaningful digital-health engagement across diverse chronic disease-care environments.

Public health, digital divide, and LMIC implementation challenges

From a public-health perspective, digital technologies have expanded healthcare delivery beyond individual patient management to include surveillance, prevention, continuity of care, remote monitoring, and health-system strengthening [1,6]. In India, initiatives such as the National Digital Health Mission and Ayushman Bharat Digital Mission reflect ongoing efforts to improve accessibility, continuity, interoperability, and integration of healthcare services through digital platforms [6].

However, the expansion of digital infrastructure alone may not ensure the meaningful utilisation of digital-health services. In many LMIC settings, implementation continues to be influenced by disparities in digital capability, literacy, behavioural readiness, connectivity, and healthcare accessibility [7-9]. Older adults, socioeconomically disadvantaged populations, individuals with lower educational attainment, and residents of geographically constrained regions may experience particular challenges in engaging with digital-health systems [9].

Importantly, underutilisation may persist despite nominal access to smartphones or internet connectivity. Meaningful engagement with digital-health platforms frequently depends on additional factors, including eHealth literacy, trust in digital systems, provider support, language compatibility, and integration within routine healthcare workflows [7-10]. Fragmented digital ecosystems, limited interoperability, inadequate technical support, and variable provider training may further contribute to inconsistent utilisation patterns across healthcare settings.

These observations support the need for implementation-oriented frameworks that extend beyond simplistic access-based assessment and instead incorporate structural, behavioural, contextual, and health-system determinants of utilisation. The multidomain framework proposed in this report attempts to address this need by providing a structured and adaptable approach for evaluating barriers influencing digital-health engagement across diverse NCD-care settings in LMIC environments.

Relevance across the NCD spectrum

Although digital-health implementation has frequently been evaluated within diabetes-care settings, many determinants influencing utilisation are applicable across the broader spectrum of chronic NCD care. Conditions such as hypertension, cardiovascular disease, chronic respiratory disease, and multimorbidity similarly require long-term follow-up, continuity of care, behavioural modification, medication adherence, and sustained patient engagement.

In hypertension and cardiovascular disease, digital-health approaches, including remote blood-pressure monitoring, telemonitoring systems, medication reminders, and teleconsultation platforms, may support continuity of care and secondary prevention strategies [16]. Similarly, in chronic respiratory disease, remote symptom monitoring and digital follow-up systems may help improve accessibility and reduce barriers related to travel and healthcare availability.

The relevance of digitally enabled care pathways is particularly important in LMIC settings where chronic disease burden frequently coexists with workforce shortages, fragmented follow-up systems, and substantial geographical barriers to healthcare access. Under such conditions, structured assessment of barriers influencing digital-health utilisation may help support more context-sensitive implementation strategies across diverse chronic disease-care environments.

The framework proposed in this report is therefore intended not as a disease-specific model but as an adaptable implementation-oriented approach capable of supporting assessment across multiple chronic NCD-care settings. Illustrative applications of the framework across selected NCD settings are summarised in Table 3.

**Table 3:** Illustrative Application of the Proposed Framework Across Selected Non-communicable Disease Settings Note: Key implementation challenges are illustrative examples intended to demonstrate potential barriers to digital-health utilisation and do not represent an exhaustive list. Additional factors may include service availability, patient preferences, usability and accessibility concerns, provider engagement, financial or connectivity constraints, discontinuation of use, clinical appropriateness, and other context-specific implementation barriers. Abbreviations: NCD, non-communicable disease; SMS, short message service.

NCD Setting	Potential Digital-Health Applications	Key Implementation Challenges
Diabetes mellitus	Teleconsultation, glucose-monitoring applications, medication reminders, digital education platforms	Digital literacy, sustained behavioural engagement, treatment adherence
Hypertension	Remote blood-pressure monitoring, SMS reminders, tele-follow-up systems	Long-term adherence, affordability, intermittent connectivity
Cardiovascular disease	Telemonitoring, cardiac rehabilitation support, medication adherence platforms	Workflow integration, provider engagement, continuity of follow-up
Chronic respiratory disease	Remote symptom monitoring, virtual follow-up, patient education systems	Technological familiarity, accessibility, geographical barriers
Multimorbidity care	Integrated teleconsultation and chronic disease-management platforms	Coordination complexity, fragmented care pathways, digital fatigue

Strengths and practical contributions of the framework

The findings from existing literature, together with practical implementation experience, support the need for digital-health strategies that extend beyond technology availability alone and instead incorporate behavioural, literacy-related, structural, and health-system determinants of utilisation. The framework presented in this report attempts to address this need through a multidomain and implementation-oriented assessment approach applicable across diverse NCD-care settings.

An important strength of the framework lies in its operational adaptability. By integrating the assessment of structural access, individual capability, behavioural readiness, and health-system integration within a single model, the framework provides a structured method for identifying context-specific barriers influencing digital-health utilisation. The inclusion of a stepwise workflow, operational checklist, and illustrative scoring template may additionally support reproducibility and facilitate practical implementation within routine clinical and public-health settings.

The framework may also support comparability across digital-health implementation studies conducted in different healthcare environments and chronic disease settings. Structured assessment of utilisation determinants may help clinicians, researchers, programme managers, and policymakers identify vulnerable populations, prioritise implementation gaps, and design context-sensitive interventions, including digital-literacy initiatives, workflow integration, provider training, language adaptation, and strengthening of telehealth support systems.

Importantly, the framework is intended primarily as a practical implementation-oriented tool rather than a rigid prescriptive model or disease-specific predictive algorithm. Its flexibility across different healthcare contexts may therefore represent an important advantage, particularly in heterogeneous LMIC settings where digital-health readiness, healthcare infrastructure, and population characteristics may vary substantially.

Limitations

Several limitations of the present framework should be acknowledged. The framework is primarily implementation-oriented and was developed to support structured assessment of digital-health utilisation across routine NCD-care settings rather than to function as a disease-specific predictive model. Although informed by operational experience within a tertiary-care outpatient setting, its broader applicability across different healthcare environments and population groups warrants further evaluation. Formal stakeholder engagement, Delphi-based consensus development, psychometric validation, and external validation were beyond the scope of the present report and represent important priorities for future research.

The operational indicators and illustrative scoring approaches included within the framework are intended to provide flexible guidance for structured assessment and may require contextual adaptation according to local healthcare infrastructure, population characteristics, and implementation priorities. Variability in digital-health readiness, healthcare-system capacity, and sociocultural context across LMIC settings may additionally influence implementation patterns and operational interpretation.

The present report also does not include prospective evaluation of framework-guided interventions or long-term implementation outcomes. Future implementation studies conducted across diverse NCD-care environments may help refine operational components of the framework and further assess its practical utility within routine healthcare settings.

Despite these limitations, the framework attempts to provide a pragmatic and adaptable approach for systematic evaluation of barriers influencing digital-health utilisation in resource-constrained healthcare environments.

## Conclusions

Digital-health technologies have considerable potential to strengthen management of NCDs in LMICs; however, meaningful utilisation remains inconsistent despite expanding digital infrastructure and increasing availability of telemedicine and mobile-health platforms. Underutilisation reflects a complex interaction of structural, behavioural, literacy-related, and health-system determinants rather than limitations in technology alone. The multidomain framework presented in this report provides a practical and implementation-oriented approach for systematic assessment of digital-health utilisation across diverse NCD-care settings. By incorporating structural access, individual capability, behavioural readiness, and health-system integration within a single operational framework, the proposed approach supports a broader evaluation of barriers influencing engagement with digital-health services in routine clinical and public-health environments.

The framework was informed by practical outpatient implementation experience within an LMIC healthcare setting and is intended to remain adaptable across a broad spectrum of chronic NCD conditions, including diabetes, hypertension, cardiovascular disease, chronic respiratory disease, and multimorbidity care pathways. Strengthening digital-health implementation in LMICs will require approaches that extend beyond technology deployment alone and instead address issues related to digital literacy, contextual adaptation, provider engagement, and integration into routine healthcare workflows. By facilitating the systematic identification of determinants influencing digital-health utilisation, the framework may help inform implementation strategies such as training, workflow adaptation, language localisation, and technical support, with the potential to improve implementation processes, service delivery, and patient engagement within chronic disease care. The proposed framework may assist researchers, clinicians, programme managers, and policymakers in evaluating barriers to digital-health utilisation and supporting development of more context-sensitive and scalable digital-health implementation strategies within resource-constrained healthcare environments.
